# L-Stepholidine rescues memory deficit and synaptic plasticity in models of Alzheimer's disease via activating dopamine D1 receptor/PKA signaling pathway

**DOI:** 10.1038/cddis.2015.315

**Published:** 2015-11-05

**Authors:** J-R Hao, N Sun, L Lei, X-Y Li, B Yao, K Sun, R Hu, X Zhang, X-D Shi, C Gao

**Affiliations:** 1 Jiangsu Key Laboratory of Anesthesiology, Xuzhou Medical College, Jiangsu 221004, China; 2 Jiangsu Key Laboratory of Anesthesia and Analgesia Application, Xuzhou Medical College, Jiangsu 221004, China

## Abstract

It is accepted that amyloid β-derived diffusible ligands (ADDLs) have a prominent role in triggering the early cognitive deficits that constitute Alzheimer's disease (AD). However, there is still no effective treatment for preventing or reversing the progression of the disease. Targeting *α*-amino-3-hydroxy-5-methylisoxazole-4-propionic acid (AMPA) receptor trafficking and its regulation is a new strategy for AD early treatment. Here we investigate the effect and mechanism of L-Stepholidine (L-SPD), which elicits dopamine D1-type receptor agonistic activity, while acting as D2-type receptor antagonist on cognition and synaptic plasticity in amyloid precursor protein (APP) and presenilin 1 (PS1) double-transgenic (APP/PS1) mice, and hippocampal cultures or slices treated with ADDLs. L-SPD could improve the hippocampus-dependent memory, surface expression of glutamate receptor A (GluA1)-containing AMPA receptors and spine density in hippocampus of APP/PS1 transgenic mice. L-SPD not only rescued decreased phosphorylation and surface expression of GluA1 in hippocampal cultures but also protected the long-term potentiation in hippocampal slices induced by ADDLs. Protein kinase A (PKA) agonist Sp-cAMPS or D1-type receptor agonist SKF81297 had similar effects, whereas PKA antagonist Rp-cAMPS or D1-type receptor antagonist SCH23390 abolished the effect of L-SPD on GluA1 trafficking. This was mediated mainly by PKA, which could phosphorylate serine residue at 845 of the GluA1. L-SPD may be explored as a potential therapeutic drug for AD through a mechanism that improves AMPA receptor trafficking and synaptic plasticity via activating D1/PKA signaling pathway.

Alzheimer's disease (AD) is an age-dependent neurodegenerative disorder, which likely begins with deficits in synaptic transmission in brain regions such as the hippocampus. Now it is accepted that amyloid β-derived diffusible ligands (ADDLs) have a key role in synapse dysfunction and early memory loss in AD.^1, 2^ Excitatory synaptic transmission is tightly regulated by *α*-amino-3-hydroxy-5-methylisoxazole-4-propionic acid (AMPA) receptors. Regulation of AMPA receptor trafficking in and out of excitatory synapses is important for controlling the strength of excitatory synapses in long-term potentiation (LTP), long-term depression and other forms of synaptic plasticity.^3, 4, 5^ Regulation of AMPA receptor trafficking depends on receptor subunit composition. In the hippocampus, most AMPA receptors are either glutamate receptor A (GluA)1/GluA2 (~80%) or GluA2/GluA3 (~20%) hetero-oligomers.^6, 7^ GluA1-containing receptors are delivered in an activity-dependent manner during LTP.^8^ In hippocampal CA1 pyramidal neurons, LTP involves insertion of AMPA receptors into excitatory synapses. GluA1-containing AMPA receptor phosphorylation regulates AMPA receptor exocytosis and subsequent insertion at synapses.^3, 9^

Recent studies have shown that Aβ oligomers could alter AMPA receptor trafficking by modulating relevant protein kinases or protein phosphatases.^10^ However, the mechanisms through which ADDLs modify AMPA receptor trafficking and synaptic plasticity, as well as cognitive deficits, are not fully known.

Protein kinase A (PKA) activation strongly increases exocytosis of AMPA receptors by direct phosphorylation of AMPA receptors.^11^ Dopamine receptors have been grouped into two families, D1 type and D2 type. The D1 type comprises D1 and D5 receptor subtypes, which couple to G*α*_s_/cyclic AMP (cAMP), leading to activation of PKA, whereas the D2 type comprises D2, D3 and D4 receptor subtypes, which couple to G*α*_i_/cAMP, leading to inactivation of PKA. Our previous works have demonstrated that brief activation of D1-type receptor accelerates GluA1 externalization at extrasynaptic sites through a PKA-dependent pathway in hippocampal pyramidal neurons.^12^ Another study has shown that activation of D1-type dopamine receptors protects neurons from synapse dysfunction induced by amyloid-β oligomers.^13^ Thus, L-Stepholidine (L-SPD), one of the active ingredients of the Chinese herb *Stephania intermedia* belonging to the tetrahydroprotoberberines (THPBs), which elicits dopamine D1-type receptor agonistic activity, while acting as D2-type receptor antagonist,^14, 15^ may have potential therapeutic effect on AD by improving AMPA receptor trafficking and synaptic plasticity.

In the present study, we demonstrated for the first time that L-SPD improved the impaired memory that was correlated with decreased phosphorylation and surface expression of GluA1-containing AMPA receptors, as well as spine loss in amyloid precursor protein (APP) and presenilin 1 (PS1) double-transgenic (APP/PS1) mice. In addition, L-SPD prevented ADDL-induced dysfunction of AMPA receptor trafficking in cultured hippocampal neurons and the inhibition of LTP in hippocampal slices through activation of D1/PKA signaling pathway.

## Results

### L-SPD improves impaired learning and memory in APP/PS1 transgenic mice

**Figure 1:**
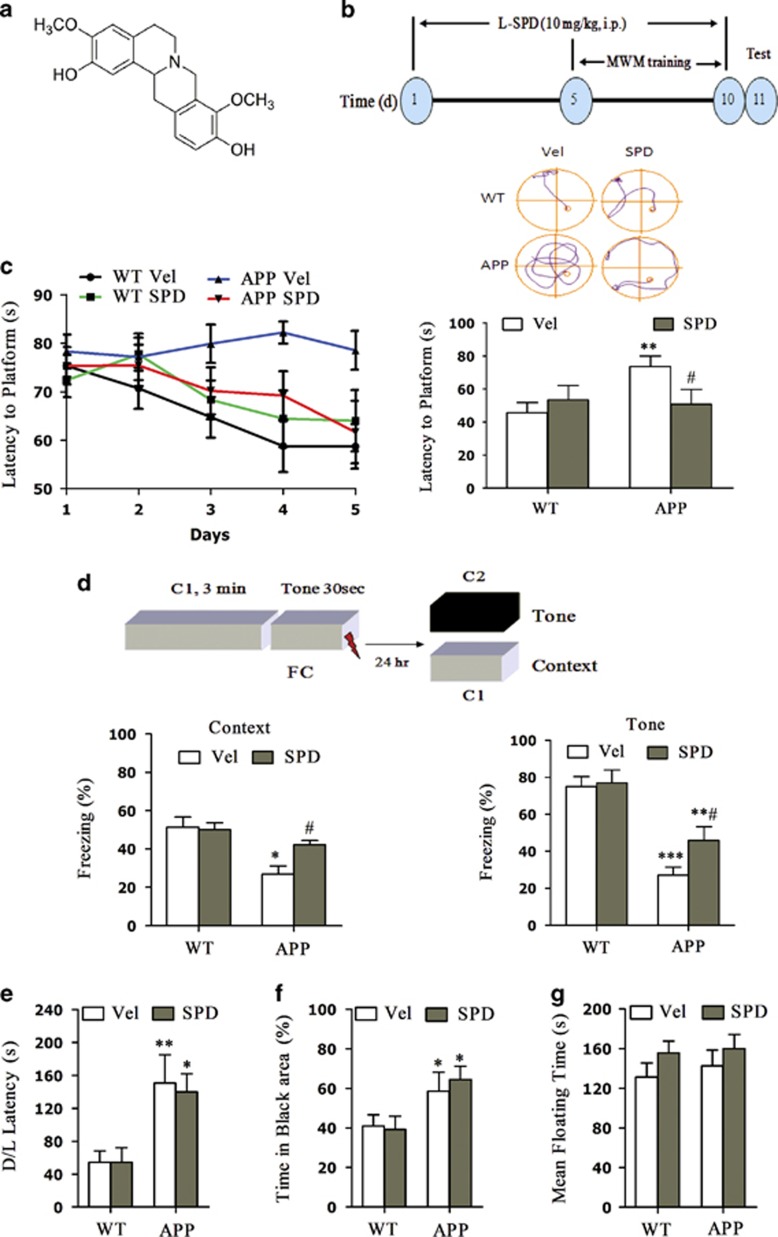
L-SPD improves impaired learning and memory in APP/PS1 mice. (**a**) Chemical structure of L-SPD. (**b**) Schedule of test. (**c**) APP/PS1 mice (APP) injected with L-SPD (10 mg/kg, i.p.) spent less time on reaching the platform than that of DMSO vehicle (Veh) after training (*n*=16 in each group). The latency to first reach the platform was recorded 24 h after last training session. Representative routes to reach the platform are on the top. APP mice injected with vehicle spent more time on reaching the platform compared with WT mice injected with vehicle, whereas mice injected with L-SPD spent less time than that of the vehicle (*n*=11 in WT/Vel group, *n*=10 in WT/SPD group, *n*=14 in APP/Vel group, *n*=9 in APP/SPD group). (**d**) L-SPD significantly improved the impaired context-dependent fear memory and partly improved the impaired tone-dependent fear memory in APP/PS1 mice compared with WT mice (*n*=8 in each group). (**e** and **f**) L-SPD did not affect anxiety-like behavior. D/L test showed increased latency to exit the dark area **(e)** and the percentage of time spent in dark area (**f**) in APP/PS1 mice compared with WT mice treated with vehicle. L-SPD did not affect the latency and time spent on black area (*n*=7 in APP/Vel group, *n*=8 in other groups). (**g**) L-SPD did not affect depression-like behavior. Forced swim test showing similar time spent on floating in APP/PS1 mice compared with WT mice treated with vehicle. L-SPD did not affect the mean floating time (*n*=13 in APP/Vel group, *n*=15 in other groups). *P<0.05, **P<0.01, ***P<0.001 *versus* corresponding WT group; #*P*<0.05 *versus* APP/vehicle group. Data are represented as mean±S.E.M

Spatial learning and memory is usually impaired in the mouse model of AD.^16^ We first studied the effect of L-SPD on learning and memory in APP/PS1 (APP) mice. L-SPD belongs to THPBs (Figure 1a). In the pilot experiment, L-SPD itself (3, 6 and 10 mg/kg, i.p.) had no effect on locomotor activity in WT mice injected for 10 days (data not shown). Then, 9-month-old littermate APP/PS1 and WT mice were injected with L-SPD (10 mg/kg, i.p.) or dimethyl sulfoxide (DMSO) vehicle (Vel, 10%, i.p.) for 10 days (Figure 1b). The dosage of 10 mg/kg was chosen for the subsequent tests based on other reports.^14, 15^ All animals were trained in the Morris water maze (MWM) test during the last 5 days (Groups: F_3_=8.836, *P*<0.001; Days: F_4_=3.3991, *P*=0.004; Figure 1c). After the second trial day, APP mice injected with vehicle spent more time on reaching the platform compared with WT mice (*P*<0.001), whereas APP mice injected with L-SPD spent less time than that of the vehicle on both training days (*P*=0.001) and the latency, to first reach the platform on test day (*P*=0.033; Figure 1c). Similar protective results were obtained in fear conditioning test (Context: F_(3,28)_=7.811, *P*=0.01; Tone: F_(3,28)_=12.560, *P*<0.001; Figure 1d). Both context- and tone-dependent memory were impaired in APP/PS1 mice. L-SPD significantly rescued the impaired context-dependent fear memory (*P*=0.045, compared with vehicle group of APP/PS1 mice) and partly improved tone-dependent fear memory (*P*=0.005, compared with L-SPD group of WT mice; *P*=0.037, compared with vehicle group of APP/PS1 mice) in APP/PS1 mice. These results demonstrate that L-SPD can improve hippocampus-dependent memory loss.

We next examined whether L-SPD could affect anxiety or depression-like behavior. These behaviors were evaluated by open field and forced swim test, respectively. APP mice showed an anxiety-like behavior (Latency: F_(3,27)_=5.638, *P*=0.004; Time in black area: F_(3,27)_=3.859, *P*=0.020; Figures 1e and f). L-SPD did not affect the latency (*P*=0.739) and time exploring in the black area (*P*=0.567). Forced swim test showed similar time spent on floating in APP/PS1 mice compared with WT mice. L-SPD did not affect the mean floating time (F_(3,55)_=0.786, *P*=0.507; Figure 1g). No depressive-like behavior was detected in APP/PS1 mice in the current study and L-SPD itself can not induce depressive-like behavior. These results demonstrate a specific role of L-SPD in improving learning and memory in APP/PS1 mice.

### L-SPD rescues the surface expression of GluA1-containing AMPA receptors and spine density in the hippocampus of APP/PS1 transgenic mice

**Figure 2:**
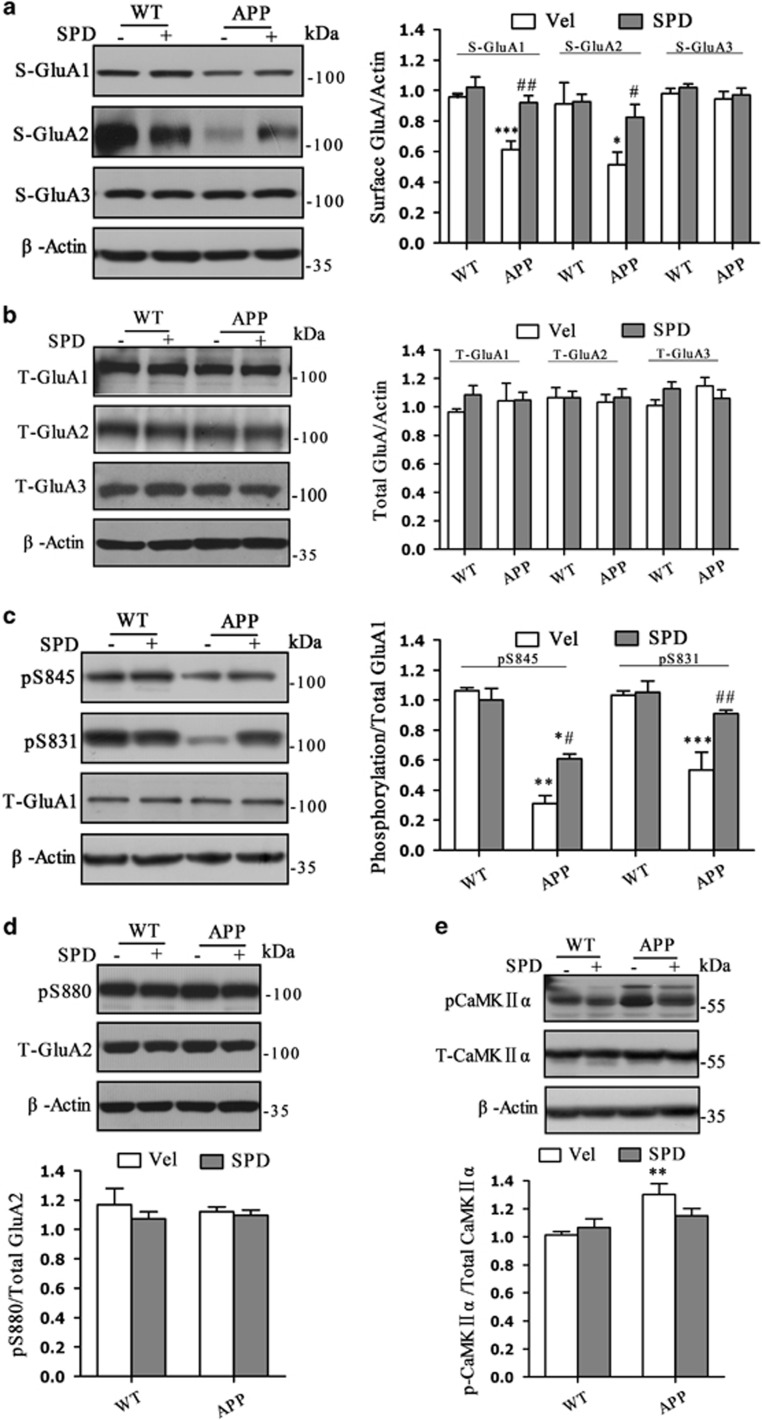
L-SPD rescues the decreased phosphorylation and surface expression of GluA1-containing AMPA receptors in the hippocampus of APP/PS1 mice. (**a**) Surface expression of GluA1 and GluA2 subunits were decreased, whereas S-GluA3 expression was not changed in APP/PS1 mice treated with vehicle. L-SPD significantly improved the levels of surface expression of GluA1 and GluA2 in APP/PS1 mice. (GluA1: *n*=4 in each group, GluA2: *n*=5 in each group, GluA3: *n*=4 in each group). (**b**) Total expression of GluA1, GluA2 and GluA3 subunits were not changed (*n*=4 in each group). (**c**) Phosphorylated levels of both pS845 and pS831 of GluA1 were decreased in APP/PS1 mice treated with vehicle. L-SPD could improve the levels of both pS845 and pS831 (*n*=4 in each group). (**d**) Phosphorylated levels of pS880 of GluA2 was not changed and L-SPD had no effect on pS880 in APP/PS1 mice (*n*=3 in each group). (**e**) Phosphorylated levels of CaMKIIα was increased in APP /PS1 mice treated with vehicle. L-SPD had no effect on p-CaMKIIα (*n*=6 in each group). *P<0.05, **P<0.01, ***P<0.001 *versus* corresponding WT group; ^#^*P*<0.05, ^##^*P*<0.01 *versus* APP/vehicle group. Data are represented as mean±S.E.M

To further investigate potential mechanisms underlying cognitive dysfunction between APP/PS1 and WT mice, we measured the expression of surface GluA1–3 (S-GluA1: F_(3,12)_=12.575, *P*=0.001; S-GluA2: F_(3,16)_=4.063, *P*= 0.025; S-GluA3: F_(3,12)_=0.608, *P*=0.622; Figure 2a) and total GluA1–3 (T-GluA1: F_(3,12)_=0.451, *P*=0.6147; T-GluA2: F_(3,12)_=0.080, *P*=0.970; T-GluA3: F_(3,12)_=1.350, *P*=0.281; Figure 2b) in the hippocampus. Surface expression of GluA1 and GluA2 subunits were decreased in APP/PS1 mice (S-GluA1: *P*<0.001; S-GluA2: *P*=0.010), whereas the S-GluA3 expression were not changed. L-SPD significantly rescued the levels of S-GluA1/GluA2 in APP/PS1 mice (S-GluA1: *P*=0.001; S-GluA2: *P*=0.035; Figure 2a). Total expressions of GluA1–3 were not changed (Figure 2b).

Surface expression of GluA1-containing AMPA receptors is correlated with its phosphorylated level, especially at Serine 845 (S845) and S831 of GluA1.^12, 17, 18^ Next, we detected phosphorylated (pS845 and pS831) levels of GluA1 (pS845: *F*_(3,12)_=47.866, *P*<0.001; pS831: *F*_(3,12)_=10.937, *P*<0.001). Both pS845 (*P*=0.001) and pS831 (*P*<0.001) of GluA1 were decreased in APP/PS1 mice. L-SPD significantly improved the phosphorylated levels in APP/PS1 mice (pS845: *P*=0.020; pS831: *P*=0.003; Figure 2c). By contrast, phosphorylation of S880 in GluA2 was not changed in APP/PS1 mice (F_(3,12)_=0.701, *P*=0.571; Figure 2d). However, the level of activated calcium/calmodulin-dependent protein kinase II (pCaMKIIα), which can phosphorylate at S831 in GluA1, was increased in APP/PS1 mice (F_(3,19)_=4.742, *P*=0.012; Figure 2e). This may exclude the effect of CaMKII on decreased phosphorylation at S831.

**Figure 3:**
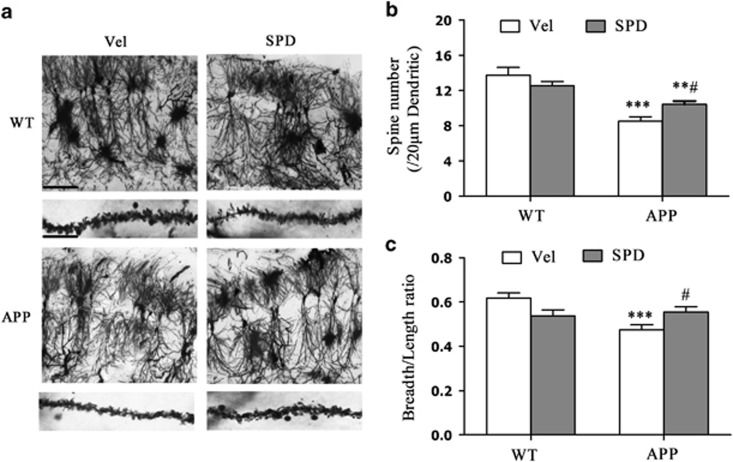
L-SPD improves spine density in the hippocampus of APP/PS1 mice. (**a**) Golgi staining of the hippocampal CA1 area (*n*=6 in each group). Scale bars= 70 μm (top panel); 15 μm (bottom panel). (**b**) Quantitative analysis of spine density. The spine number was decreased in the APP/PS1 mice treated with vehicle. L-SPD partly rescued the impaired spine density (*n*=20 in each group). (**c**) Quantitative analysis of breadth-to-length ratio. The ratio was decreased in the APP/PS1 mice treated with vehicle. L-SPD significantly improved the impaired ratio (*n*=51 in each group). **P<0.01, ***P<0.001 *versus* corresponding WT group; ^#^*P*<0.05 *versus* APP/vehicle group. Data are represented as mean±S.E.M

In addition, we calculated the spine numbers in hippocampal CA1 area as revealed by Golgi staining (*F*_(3,76)_=16.122, *P*<0.001; Figures 3a and b). APP/PS1 mice showed a decrease in the number of dendritic spines (*P*<0.001). L-SPD could partly improve the spine density in APP/PS1 mice (*P*=0.024). To better understand the morphology of dendritic spines, we calculated the breadth-to-length ratio for each spine (*F*_(3,200)_=5.663, *P*<0.001; Figure 3c). The ratio was decreased in APP/PS1 mice (*P*<0.001). After L-SPD treatment, the breadth-to-length ratio was recovered (*P*=0.022).

Together, these findings show that reduced surface expression of GluA1/GluA2 and spine density in the hippocampus are relevant to memory loss in APP/PS1 mice. L-SPD may have potential therapeutic effect on the mouse model of AD.

### Activation of D1/PKA rescues decreased surface expression of GluA1-containing AMPA receptors induced by ADDLs in cultured hippocampal neurons

**Figure 4:**
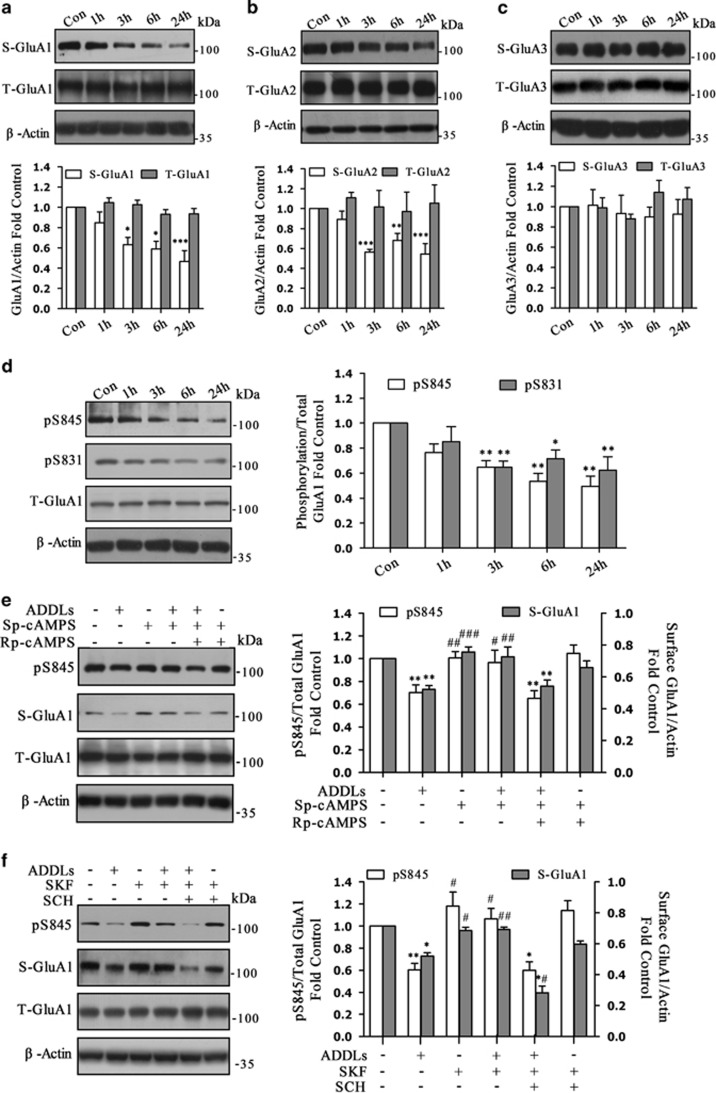
Activation of D1/PKA rescues the decreased GluA1-containing AMPA receptor surface expression induced by ADDLs in cultured hippocampal neurons. (**a**–**c**) Surface expression of GluA1 (S-GluA1) and GluA2 (S-GluA2) were significantly decreased after treating with ADDLs (500 nM) for 3–24 h, whereas surface expression of GluA3 (S-GluA3) was not changed. Total expression of GluA1-3 (T-GluA) were not changed. (GluA1: *n*=5 in each group, GluA2: *n*=4 in each group, GluA3: *n*=4 in each group). (**d**) Phosphorylated levels of GluA1 at Serine 845 (pS845) and Serine 831 (pS831) were decreased after treating with ADDLs (pS845: *n*=9 in each group, pS831: *n*=9 in each group). (**e**) Pretreatment with PKA activator Sp-cAMPS (10 μM) rescued the decreased pS845 and surface expression of GluA1 induced by ADDLs, whereas the PKA inhibitor Rp-cAMPS (10 μM) reversed these effects. Sp-cAMPS or Rp-cAMPS was added 10 min before ADDLs (*n*=4 in each group). (**f**) Pretreatment with D1-type receptor agonist SKF 81297 (SKF, 3 μM) rescued the decreased pS845 and surface expression of GluA1 induced by ADDLs, whereas the antagonist SCH 23390 (SCH, 10 μM) reversed these effects. SKF 81297 or SCH 23390 was added 15 min before ADDLs (*n*=6 in each group). *P<0.05, **P<0.01, ***P<0.001 *versus* corresponding control group (Con); ^#^*P*<0.05, ^##^*P*<0.01, ^###^*P*<0.001 *versus* corresponding ADDL group. Data are represented as mean±S.E.M

To explore the cellular and molecular mechanisms of the protective effect of L-SPD on AMPA receptor trafficking, we next measured the surface expression of AMPA receptor subunits in response to ADDLs at various times in cultured hippocampal neurons. Primary neuronal cultures were treated with freshly prepared 500 nM ADDLs (Supplementary Figure S1) for 1, 3, 6 and 24 h. Next, surface proteins were extracted and surface expressions of GluA1–3 (S-GluA) were detected. ADDLs significantly decreased membrane GluA1 and GluA2 protein levels after a 3-h treatment (S-GluA1: F_(4,20)_=6.749, *P*=0.001; S-GluA2: F_(4,15)_=8.603, *P*=0.001) but had no effect on GluA3 surface expression (F_(4,10)_=0.159, *P*=0.955; Figures 4a–c). Under the same conditions, total amount of GluA1–3 (T-GluA) were not changed (T-GluA1: F_(4,20)_=1.595, *P*=0.214; T-GluA2: F_(4,20)_=0.137, *P*=0.966; T-GluA3: F_(4,15)_=1.234, *P*=0.338; Figures 4a and c). Similar results were obtained by using biotinylation method (Supplementary Figure S2). Given that there is no change in the surface expression of GluA3 subunit, these results suggest that ADDLs mainly decrease surface expression of GluA1-containing AMPA receptors in cultured hippocampal neurons.

The PKA-mediated phosphorylation of the S845 residue of the GluA1 subunit correlates with the externalization of GluA1-containing AMPA receptors on the extrasynaptic membrane, whereas the CaMKII-mediated phosphorylation of the S831 is important for subsequent retention at synapses.^12, 18, 19^ To test the initial role of pS845 in GluA1 trafficking, we next analyzed the pS845 level. ADDLs induced a significant decrease in pS845 after incubation for 3 h (F_(4,40)_=11.716, *P*<0.001; Figure 4d). Similar results were found in pS831 (F_(4,20)_=3.712, *P*=0.020; Figure 4d).

Next, we explored whether PKA-mediated phosphorylation of GluA1 S845 was involved in ADDL-induced decrease of GluA1 phosphorylation and cell surface expression. Pretreatment with Sp-cAMPS (10 μM, 15 min), a membrane-permeable PKA activator rescued both the decreased pS845 and surface expression of GluA1 induced by ADDLs for 3 h (pS845: F_(5,24)_=5.962, *P*=0.01; S-GluA1: F_(5,24)_=6.689, *P*<0.001; Figure 4e). Surface expression of GluA2 was also rescued (F_(5,18)_=3.495, *P*=0.022; figure not shown). These effects were abolished by Rp-cAMPS (10 μM), a membrane-permeable PKA inhibitor, when co-administered with Sp-cAMPS (Figure 4e), implicating an important role of PKA signaling pathway in ADDL-mediated decreased surface expression of GluA1-containing AMPA receptors.

D1-type dopamine receptors are positively coupled to PKA. We suggested that activation of D1-type receptor prevented loss of GluA1 on membrane correlating with preservation of pS845 residue of the GluA1. When hippocampal neurons were pre-incubated with SKF 81297 (3 μM, 15 min), a D1-type receptor agonist, followed by a 3-h exposure to ADDLs (S-GluA1: F_(5,18)_=47.222, *P*<0.001; pS845: F_(5,27)_=9.727, *P*<0.001), there was no decrease in surface (*P*=0.999) and pS845 (*P*=0.560) expression of GluA1 compared with control cells (Figure 4f). However, SCH 23390, a selective D1-type receptor antagonist, abolished the protective effects of SKF 81297 on S-GluA1 (*P*=0.039) and pS845 (*P*<0.001) expression (Figure 4f), confirming the specific involvement of activation of D1-type dopamine receptor in preservation of expressions of surface and pS845 of GluA1 induced by ADDLs.

### L-SPD prevents ADDL-induced loss of surface expression of GluA1 through activation of D1/PKA in cultured hippocampal neurons

**Figure 5:**
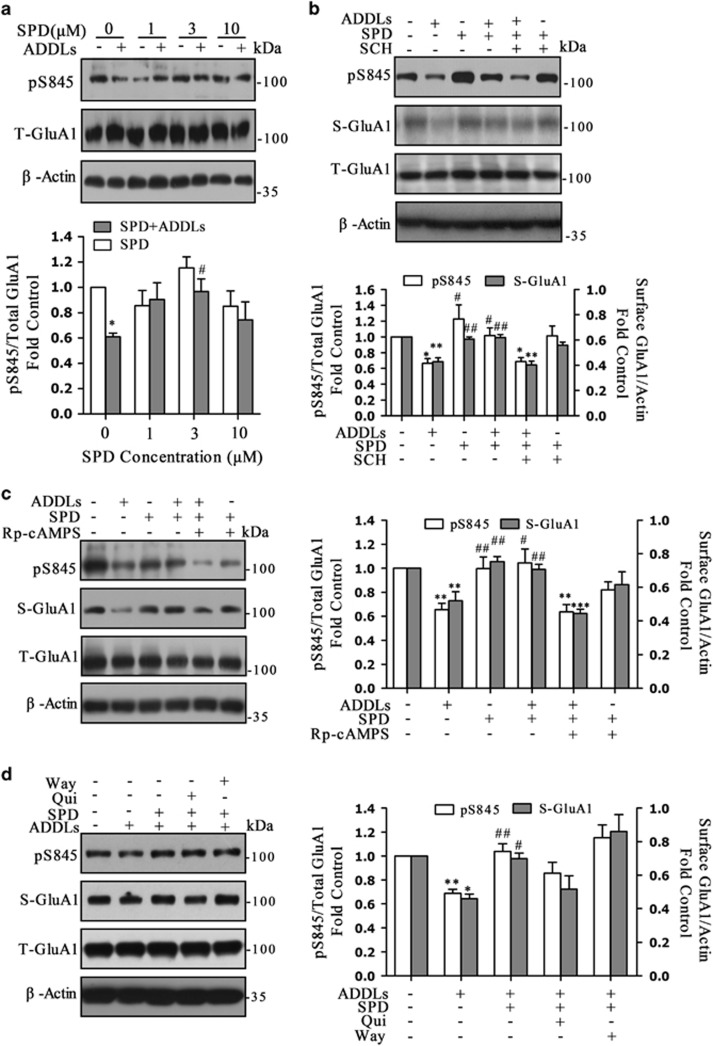
L-SPD prevents ADDL-induced loss of surface expression of GluA1 through activation of D1/PKA signal pathway in cultured hippocampal neurons. (**a**) Pretreatment with L-SPD (3 μM, 15 min) rescued pS845 of GluA1, for which phosphorylation was decreased induced by ADDLs. There was a trend toward increased pS845 under higher (10 μM) or lower (1 μM) concentration of L-SPD (*n*=8 in each group). *P<0.05 *versus* control group without L-SPD; #*P*<0.05 *versus* ADDLs group without L-SPD (*n*=4 in each group). (**b**) L-SPD (3 μM) rescued both the decreased pS845 and surface expression of GluA1 induced by ADDLs, whereas SCH 23390 (SCH, 10 μM) abolished these effects (*n*=4 in each group). (**c**) Rp-cAMPS (10 μM) abolished the effects of L-SPD (3 μM) on pS845 (*n*=5 in each group) and surface expression of GluA1 (*n*=4 in each group). (**d**) Co-administration L-SPD (3 μM) with D2 receptor agonist Quinpirole hydrochloride (Qui, 1 μM) or 5-HT_A_ receptor antagonist WAY-100635 maleate salt (Way, 1 μM) had no significant effect on the role of L-SPD in S-GluA1 expression and pS845 level (*n*=4 in each group). *P<0.05, **P<0.01, ***P<0.001 *versus* corresponding control group; ^#^*P*<0.05, ^##^*P*<0.01 *versus* corresponding ADDL group. Data are represented as mean±S.E.M

Pharmacological studies have revealed that L-SPD elicits D1 agonistic activity, a D2 receptor antagonist. It may protect neurons from synapse dysfunction induced by ADDLs through activation of D1/PKA signaling pathway. To this end, hippocampal neurons were first pre-incubated with different concentration of L-SPD (0, 1, 3 and 10 μM) for 15 min or followed by 3 h with ADDLs. As expected, 3 μM L-SPD significantly rescued the decreased pS845 of GluA1 induced by ADDLs (F_(7,40)_=2.741, *P*=0.020; Figure 5a). There was a trend toward protective effect under 1 or 10 μM. SCH 23390 (10 μM) abolished the effect of L-SPD (3 μM) on pS845 (F_(5,18)_=4.384, *P*=0.009) and surface expression of GluA1 (F_(5,18)_=18.740, *P*<0.001; Figure 5b), which indicated that L-SPD exerted its effect mainly through D1-type dopamine receptors.

Next, we checked whether PKA inhibitor would prevent the effect of L-SPD. Cultures were pre-incubated for 10 min with Rp-cAMPS (10 μM) or combined with L-SPD (3 μM) followed by ADDLs for 3 h (pS845: F_(5,30)_=6.423, *P*<0.001; S-GluA1: F_(5,18)_=8.017, *P*<0.001). Rp-cAMPS abolished the effect of L-SPD on pS845 (*P*=0.001), surface expression of GluA1 (*P*<0.001; Figure 5c), as well as surface expression of GluA2 (*P*=0.037; figure not shown).

L-SPD also had modest affinity for D2 receptor and serotonergic 5-HT_1A_ receptor.^15^ When cultures were co-administered L-SPD with D2 receptor agonist Quinpirole hydrochloride (Qui, 1 μM) or 5-HT_1A_ receptor antagonist WAY-100635 maleate salt (Way, 1 μM), neither could reverse the protective effects of L-SPD on S-GluA1 (Qui: *P*=0.087; Way: *P*=0.269) and pS845 (Qui: *P*=0.501; Way: *P*=0.731) induced by ADDLs (S-GluA1: F_(4,10)_ =7.164, *P*=0.005; pS845: F_(4,15)_ =6.494, *P=*0.003; Figure 5d). These results suggest that L-SPD prevent ADDL-induced dephosphorylation and loss of surface expression of GluA1-containing AMPA receptors mainly through activation of D1/PKA signaling pathway.

### L-SPD rescues the decreased externalization and synaptic expression of GluA1-containing AMPA receptors in cultured hippocampal neurons as well as LTP impairment in hippocampal slices induced by ADDLs

**Figure 6:**
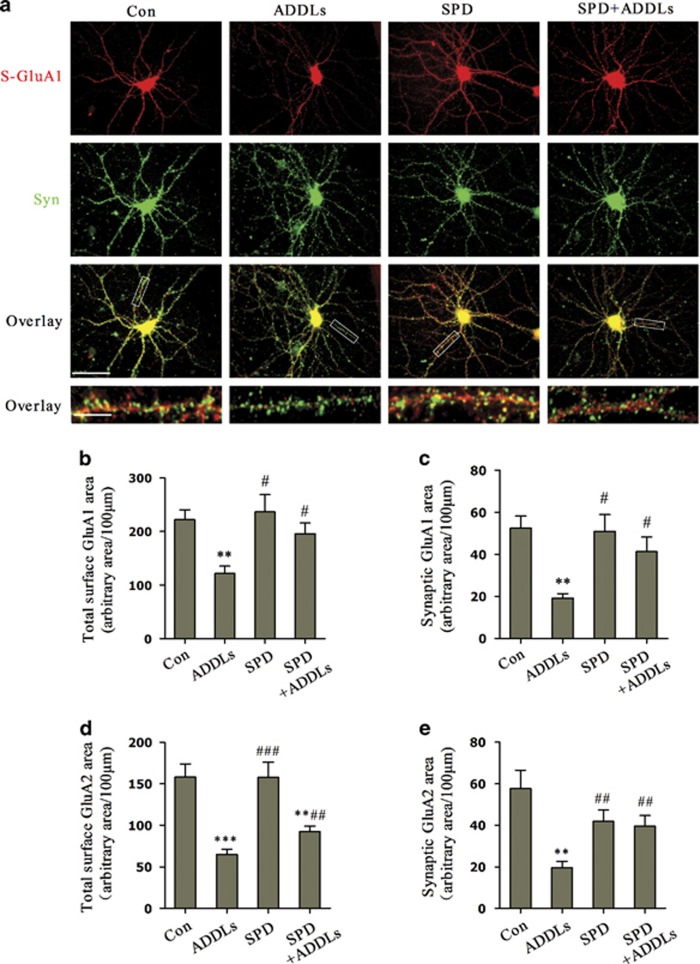
L-SPD rescues the decreased externalization and synaptic expression of GluA1/GluA2 in cultured hippocampal neurons induced by ADDLs. (**a**) Double staining against N-terminal S-GluA1 (S-GluA1, red) and synaptophysin (Syn, green) showed that pretreatment with L-SPD (3 μM) rescued both the decreased total surface and synaptic (yellow) expression of GluA1 induced by ADDLs (500 nM, 3 h). No change in synaptophysin was observed. Scale bars=100 μm (top three panels); 15 μm (bottom panel). (**b**) Quantitative analysis of total S-GluA1 area. (**c**) Quantitative analysis of synaptic GluA1 area. Error bars indicate S.E.M. from at least three independent experiments with 25 cells imaged per experimental condition in each experiment. (**d**) Quantitative analysis of total S-GluA2 area. (**e**) Quantitative analysis of synaptic GluA2 area. Error bars indicate S.E.M. from at least three independent experiments with 27 cells imaged per experimental condition in each experiment. **P<0.01, ***P<0.001 *versus* control group (Con); ^#^*P*<0.05, ^##^*P*<0.01, ^###^*P*<0.001 *versus* ADDLs group

To study the cellular and molecular mechanisms of the protective effect of L-SPD on AMPA receptor trafficking, cultured hippocampal neurons were double stained with anti-N-terminal GluA1 ( S-GluA1) and synaptophysin (Syn, presynaptic marker; S-GluA1: F_(3,96)_=6.324, *P*=0.001; synaptic GluA1: F_(3,96)_=5.203, *P*=0.002; Figures 6a–c). Pretreatment with L-SPD (3 μM, 15 min) rescued both the decreased surface and synaptic expression of GluA1 induced by ADDLs (500 nM, 3 h; S-GluA1: *P*=0.025; synaptic GluA1: *P*=0.026; Figures 6a–c). Similar results were obtained in surface and synaptic expression of GluA2 (S-GluA2: F_(3,101)_=14.130, *P*<0.001; synaptic GluA2: F_(3,101)_=6.627, *P*<0.001; Figures 6d and e). Extrasynaptic insertion of AMPA receptors via phosphorylation events is the first step of AMPA receptor trafficking.^18^ These results demonstrate that L-SPD can improve GluA1/GluA2 externalization to extrasynaptic membrane and subsequent insertion at postsynaptic area.

**Figure 7:**
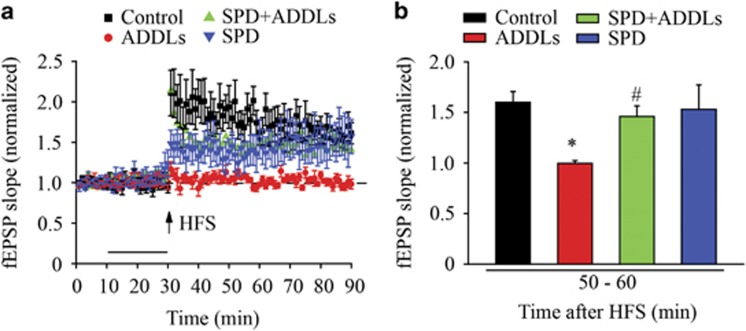
L-SPD rescues LTP impairment in hippocampal slices induced by ADDLs. (**a**) Delivery of high-frequency stimulation (HFS, 100 Hz) induced LTP in the CA1 of the hippocampus. Exposure of ADDLs (500 nM) inhibited LTP induction. Pre-incubation of L-SPD (3 μM) significantly improved the inhibition of LTP induced by ADDLs. L-SPD itself could also induce LTP under HFS. (**b**) Analysis of LTP responded at 60 min after HFS (*n*=6 in each group). *P<0.05 *versus* control group (Con); ^#^*P*<0.05 *versus* ADDLs group. Data are represented as mean±S.E.M

Next, we performed electrophysiological experiment to test the role of L-SPD in LTP. Hippocampal slices subjected to high-frequency stimulation displayed statistically significant increases in field excitatory postsynaptic potential (fEPSP) slope induced over time, lasting >60 min (F_(3,20)_=3.671, *P*=0.03; Figure 7a). Perfusion of ADDLs (500 nM) for 20 min could not induce LTP induction (*P*=0.01). L-SPD (3 μM) itself could also display LTP and significantly preserved fEPSP when it was co-perfused with ADDLs (*P* =0.027; Figure 7a and b). These results suggest that L-SPD significantly prevent LTP impairment induced by ADDLs, which links with cell surface and synaptic expression of GluA1-containing AMPA receptors.

## Discussion

Overall, we demonstrated that activation of D1-type dopamine receptor by using L-SPD could had a novel therapeutic effect on AD by improving surface expression of AMPA receptors, synaptic plasticity and hippocampus-dependent learning and memory in APP/PS1 mice. We also found that ADDLs decreased surface expression of GluA1-containing AMPA receptors by inhibiting GluA1 S845 phosphorylation and subsequent externalization, which was consistent with previous study.^13^ L-SPD prevented ADDL-induced dysfunction of AMPA receptor trafficking and impairment of LTP through activation of D1/PKA signaling pathway in hippocampal neurons. The current study extends that concept by showing important *in vivo* beneficial actions of the compound investigated.

Most excitatory synaptic transmission in the brain is mediated by AMPA receptors. Among the different subunits, GluA1 is the one whose trafficking depends on neuronal activity. The activity-dependent variation in the synaptic AMPA receptor number, also known as synaptic plasticity, is thought to be the major mechanism by which information is stored in neuronal networks.^20, 21^ It is well established that accumulation of Aβ oligomers impairs synapse function.^22, 23^ The trafficking of AMPA receptors into and out of synapses is highly dynamic and is regulated by various posttranslational modifications such as phosphorylation that occurs on their cytoplasmic C-terminal domains. Membrane insertion of GluA1 is regulated by several phosphorylation sites including S845 and S831, which are phosphorylated by PKA and CaMKII, respectively.^24^ Phosphorylation of S845 by PKA in GluA1 subunit is the first step that contributes specifically to the recruitment of new AMPA receptors to extrasynaptic sites, a critical event for the establishment of LTP.^17^ GluA1 phosphorylation correlates with lowered thresholds for LTP induction and memory formation.^25^ In the present study, the phosphorylation of GluA1 at S845 and S831 were reduced not only in cultured hippocampal neurons induced by ADDLs but also in the hippocampus of APP/PS1 mice. LTP was also inhibited in the hippocampus treated with ADDLs. Similar results are found in other groups that Aβ oligomers reduce phosphorylation of GluA1 S845 levels, leading to a decrease of surface AMPA receptors. By contrast, Aβ oligomers do not affect the phosphorylation at S831 of GluA1.^10, 13^ This is different from our result that S831 was also decreased. We suggest that ADDLs decrease surface expression of GluA1-containing AMPA receptors by inhibiting GluA1 S845 phosphorylation and subsequent externalization. The total number of S-GluA1 is reduced, which should affect the level of subsequent phosphorylation at S831 to translocate to synaptic sites. Although S831 is phosphorylated by CaMKII, activated CaMKII (pCaMKII) level was increased in APP/PS1 mice. Furthermore, phosphorylation at S880 in GluA2 by protein kinase C was not involved in decreased surface expression of GluA1-containing AMPA receptors. These results further demonstrate that decreased phosphorylation of S845 by PKA in GluA1 subunit is a crucial step that contributes to the dysfunction of AMPA receptor trafficking in AD animal or cultures model.

D1-type dopamine receptor activation leads to increased cAMP level and increased PKA activity.^26^ PKA activation strongly increases exocytosis of AMPA receptors by direct phosphorylation of GluA1 at S845.^18^ Our previous works show that D1-type receptor activation induces phosphorylation of GluA1 and AMPA receptors insertion into extrasynaptic sites in a PKA-dependent manner.^12, 27^ One recent report finds that prolonged exposure to an enriched environment, which activates β2-adrenergic receptors and downstream cAMP/PKA signaling, facilitates signaling in the hippocampus and prevents the impairment of hippocampal synaptic plasticity by Aβ oligomers.^28^ Activation of D1/PKA signaling pathway may have potential therapeutic effect on AD early cognitive dysfunction.

In this light, L-SPD, a tetrahydroberberine alkaloid isolated from the Chinese herb *S. intermedia*,^29^ is particularly interesting. L-SPD has been shown to have high affinity for dopamine D1 and modest affinity for D2 receptors and serotonergic 5-HT_1A_ receptor.^15^ Clinical trials have implicated that L-SPD is effective in the treatment of both positive and negative syndromes in schizophrenia. It could increase the frequency of sEPSC via the activation of D1 dopamine signaling pathway in rat prelimbic cortical neurons.^30^ However, the role and mechanism of L-SPD in AD has not been studied.

In the present study, we first found that L-SPD improved impaired learning and memory including spatial memory (MWM) and associative learning (fear conditioning), reduced phosphorylation and surface expression of GluA1/GluA2, as well as the spine density in the hippocampus of APP/PS1 mice. Dendritic spine formation is correlated with learning and memory. The structural plasticity induction is in line with synaptic functional plasticity and learning behaviors.^31^ APP/PS1 mice had a decreased spine density and breadth-to-length ratio that represents thinner/longer spines, namely immature spines. Our data suggested that L-SPD rescued the decreased spine density and remodeled dendritic spine shape in the hippocampus of APP/PS1 mice. LTP was also rescued in ADDL-treated hippocampal slice. The protective effect of L-SPD was mainly through activation of D1/PKA pathway as D1-type dopamine receptor or PKA antagonist could completely block its effect induced by ADDLs in cultured hippocampal neurons. Furthermore, 5-HT_1A_ receptor was not involved in this effect. These data suggest that L-SPD may promote cognitive function by enhancing AMPA receptor trafficking and synaptic plasticity through D1/PKA signaling. Furthermore, this compound is extensively transported across the blood–brain barrier.^32^ Hence, L-SPD appears to be a potentially attractive therapeutic option for AD.

The cognitive deficits in the APP/PS1 mouse model, first described by Jankowsky *et al.*,^33^ have been well characterized. It is reported that cognitive deficits are first seen in 3-month-old mice in the Radial arm water maze spatial working memory task and are also reported in 6-month-old mice in the MWM,^34, 35^ whereas the associative learning described in fear conditioning tasks start at 6–8 months.^36^ Both MWM and fear conditioning were impaired at 9-month-old mice in the present study. Anxiety disturbances have been reported in some of the AD mouse models.^37, 38, 39, 40^ In general, it starts early at 3–6 months of APP/PS1 models. Although the depressive symptoms/behaviors are a very common comorbidity with AD, very little work has been devoted to determine the range of depressive behavioral symptoms in the commonly used mouse models of AD.^16^ A higher duration of immobility in the forced swimming assay was found in APP/PS1 mice.^40^ In the present study, we found an anxiety-like behavior in APP/PS1 mice. Although there was a trend toward increased duration of immobility in the forced swimming test, there was no significant difference between WT and APP/PS1 mice at 9 months. L-SPD had no effect on anxiety and depression disturbances, which demonstrated a specific role of L-SPD in learning and memory.

In summary, L-SPD improves the learning and memory via increasing surface expression of GluA1-containing AMPA receptors and spine density in APP/PS1 transgenic mice. L-SPD prevents ADDL-induced dysfunction of AMPA receptor trafficking in cultured hippocampal neurons and impairment of LTP in hippocampal slices via activation of D1/PKA signaling pathway. L-SPD may be explored as a potential therapeutic drug for AD.

## Materials and Methods

### Animals

Male APP/PS1 mice and WT mice (nontransgenic littermates of APP/PS1 mice) were obtained from Model Animal Research Center of Nanjing University (Nanjing, China). APP/PS1 mice expressed both a chimeric mouse/human APP (Mo/Hu APP 695swe) and a mutant human PS1 (PS1-dE9). The mice were housed in small groups with free access to water and food. They were housed in a room (22±2 °C) maintained on a 12/12 dark/light (D/L) cycle (0800–2000 h) and were used when 9 months old. All studies were approved by the Animal Care and Use Committee of Xuzhou Medical College in compliance with National Institutes of Health standards.

### Drugs

Synthetic Aβ1-42 (Sangon Biotech, Shanghai, China) was dissolved in 1,1,1,3,3,3-Hexafluoro-2-propanol (Sigma-Aldrich, St. Louis, MO, USA; 10,522-8) and prepared for ADDLs as described previously.^41^ L-SPD (PureOne Biotechnology, Shanghai, China; P0164) was dissolved in DMSO (Sigma-Aldrich; D2650) at 10% final concentration in mice (3–10 mg/kg, i.p.) or at 0.01% DMSO in cultures (3–10 μM). Sp-cAMPS (A166) and Rp-cAMPS (A165) from Sigma-Aldrich, and SKF 81297 (1447) and SCH 23390 (0925) from Tocris Bioscience (Avonmouth Bristol, UK) were dissolved in NeuroBasal medium (Invitrogen, Carlsbad, CA, USA; 21103-049).

### MWM test

The MWM was performed in a round tank (1.2 m in diameter) filled with white opaque water. The day before task, mice were habituated to the environment. During the training phase, mice were allowed to swim with the platform for 90 s or until they reached the platform monitored by Anymaze software (Stoeling, Wood Dale, IL, USA). Animals that did not reach the platform after 90 s were gently guided toward it. All animals were allowed to remain on the platform for 30 s. The training phase lasted for 5 days, four trials each day. For testing, the latency to find the platform was recorded 24 h after the last training session from a starting position different to the last starting position during the training phase.

### Fear conditioning

Contextual and tone-dependent fear conditioning was performed in an automated system (Med Associates Inc., Albans, VT, USA) and consisted of a single exposure to a context (3 min) followed by a 30-s tone (10 kHz; 75 dB SPL) and a foot shock (2 s; 0.7 mA; constant current) as described previously.^42^ Context-dependent freezing was measured 24 h after every tenth second over 180 s by two observers who were unaware of the experimental conditions, and expressed as percentage of total number of observations. Freezing to the tone was scored every fifth second in a novel context during a 30-s exposure.

### Anxiety

Anxiety-like behavior was evaluated using the D/L emergence task.^42^ A 10 × 10 cm shelter was placed in the middle of the 60 × 60 cm arena. Latency to come out from the shelter and time spent in the dark area during a 5-min test were recorded automatically by Anymaze software.

### Forced swim test

Mice were placed in an upright cylinder (20 cm diameter) filled with warm water (26 °C) up to 5 cm below its opening. Mice were observed for 6 min and the amount of time spent in an immobile posture during the last 5 min was scored.

### Hippocampal cultures and treatments

The hippocampi from embryonic day 18–20 Sprague–Dawley rats were isolated and dissociated with trypsin.^12^ Cells were plated on coverslips coated with poly-D-lysine in 24-well culture plates or 6-well culture plates and grown in NeuroBasal medium with B-27 (Invitrogen; 17504). One-half of the medium was replaced with identical medium every 4 days. Cultures were kept at 37 °C in a humidified incubator with 5% CO_2_/95% air and used for experiments after 18–21 days *in vitro*. ADDLs were prepared as described previously.^41^ After stimulation with 500 nM ADDLs in NeuroBasal medium at indicated time points, the cultures were collected in cold homogenization buffer (in mM: 50 MOPS (pH 7.4), 320 sucrose, 100 KCl, 0.5 MgCl_2_, 0.2 DTT, 50 NaF, 20 NaPPi, 20 β-glycerophosphate, 1 EDTA, 1 EGTA, 1 PNPP-Na, 1 Na_3_VO_4_, 0.5 PMSF,10 μg/ml leupeptin, 10 μg/ml aprotin, 10 μg/ml pepstatin A, 100 μg/ml benzamidine).^43^ The samples were stored at −80 °C if not used immediately and sonicated before quantification. Cytoplasmic (F1) membrane (F2) fractions were prepared by using the ProteoExtract kit (Calbiochem, Darmstadt, Germany; 539790) for subcellular proteome extraction according to the manufacturer's instructions.

### Western blotting assay

After determining the protein concentration, the lysates (20 μg/well) were subjected to SDS-polyacrylamide gel electrophoresis and subsequently blotted to polyvinylidene fluoride membranes (Bio-Rad, Hercules, CA, USA; 162-0177) and incubated at 4 °C overnight with the following primary antibodies: GluA1 (Calbiochem; PC246; 1 : 1000), GluA2 (Invitrogen; 32-0300; 1 : 1000), GluA3 (Invitrogen; 32-0400; 1 : 500), phospho GluA1 S845 (Abcam, Cambridge, MA, USA; AB76321; 1 : 1000), phospho GluA1 S831 (Abcam; AB109464; 1 : 1500), phospho GluA2 S880 (Abcam; AB52180; 1 : 1000), phospho CaMKIIα (Millipore, Bedford, MA, USA; 05-533; 1 : 1000), CaMKIIα (Santa Cruz Biotechnology, Dallas, TX, USA; sc-32288; 1 : 500) and β-actin (Santa Cruz Biotechnology; sc-47778; 1 : 2000). Membranes were then incubated with horseradish peroxidase-conjugated secondary antibodies (Beyotime Institute of Biotechnology, Jiangsu, China; goat anti-rabbit, A0208; goat anti-mouse, A0216; 1 : 1000) and immunoblotted using ECL detection system (Beyotime Institute of Biotechnology; P0018). Optical densities of immune reactive bands were measured using the NIH Image J Software (National Institutes of Health, Bethesda, MD, USA).

### Golgi staining

Animals were deeply anesthetized before being killed. The brain was fixed and stained with an FD Rapid Golgistain Kit (FD NeuroTechnologies, Columbia, MD, USA; PK401) according to the manufacturer's instructions.

### Immunocytochemistry and quantitative immunofluorescence

After stimulation, the neurons were fixed with 4% formaldehyde in PBS containing 4% sucrose for 10 min at room temperature (RT). After washing with PBS for three times, the cells were blocked with 5% normal goat serum or 5% horse serum in PBS for 1 h. Next, the cells were incubated with polyclonal antibody recognizing the extracellular N-terminal domain of GluA1 (Calbiochem; 1 : 50) or GluA2 (Invitrogen; 1 : 300) in blocking buffer for 1 h at RT and secondary antibody conjugated to Cy3 (Jackson Immunoresearch Laboratories, West Grove, PA, USA; 111-165-144; goat anti-rabbit; 1 : 250) or FITC (Jackson Immunoresearch Laboratories; 309-095-095; donkey anti-mouse; 1 : 150), respectively, for 1 h at RT. After extensive washing in PBS, cells were permeabilized with 0.3% Triton X-100 in PBS for 15 min, blocked with 5% horse serum or 5% goat serum in PBS containing 0.1% Triton X-100 for 1 h and incubated with polyclone antibody to the synaptic marker synaptophysin (Cell Signaling Technology, Beverly, MA, USA; 5461; rabbit; 1 : 250 or Millipore; MAB368; mouse; 1 : 500) at 4 °C overnight, followed by donkey anti-mouse secondary antibody conjugated to FITC (1 : 150) or goat anti-rabbit secondary antibody conjugated to Cy3 (1 : 250) for 1 h at RT. After four rinses, coverslips were mounted using PVA-DABCO (Sigma-Aldrich; 10981). Images were acquired and analyzed as described previously, using an Olympus X81 microscope, QImaging Rolera XR digital camera and MetaMorph software (Molecular Devices, Sunnyvale, CA, USA).^12, 27^

### Electrophysiology

Transverse hippocampal slices (400-μm thick) were prepared from 5- to 8-week-old mice for LTP. Slices were cut in artificial cerebrospinal fluid, which contained (in mM): 126 NaCl, 2.5 KCl, 1 MgCl_2_, 1 CaCl_2_, 1.25 KH_2_PO_4_, 26 NaHCO_3_, 20 glucose, pH 7.2, 320 mOsm, equilibrated with 95% O_2_/5% CO_2_. Slices were allowed to recover for at least 90 min before recording. Following the recovery period, slices were transferred to a submerged recording chamber mounted on an Olympus dissecting microscope. A unipolar stimulating electrode (World Precision Instruments, Bowdion, ME, USA) was placed in the Schaffer collaterals, to deliver test and conditioning stimuli. fEPSPs in CA1 were induced by test stimuli at 0.05 Hz with an intensity that elicited an fEPSP amplitude of 40~50% of maximum. To induce LTP, four consecutive trains (1 s) of stimuli at 100 Hz separated by 20 s were given.^44, 45, 46^ The field potentials were amplified using Molecular Devices 700B amplifier and digitized with Digidata 1322A (Molecular Devices). The data were sampled at 10 kHz and filtered at 2 kHz. Traces were obtained by pClamp 10.2 and analyzed using the Clampfit 10.2 (DL Naturegene Life Sciences, Newbury Park, CA, USA).

### Data analysis and statistics

Water maze data were analyzed by ANOVA with repeated measure followed by *post-hoc* Student–Newman–Keuls (SNK) multiple comparisons. Other behavior tests and biochemical data were analyzed with one-way ANOVA followed by the SNK, least-significant difference (for equal variances) or Dunnett's T3 (unequal variances) multiple comparisons. Differences were considered significant when *P*<0.05. Data are shown as the mean values±S.E.M.

## Abbreviations

AD: Alzheimer's disease
ADDL: amyloid β-derived diffusible ligand
AMPA: *α*-amino-3-hydroxy-5-methylisoxazole-4-propionic acid
APP: amyloid precursor protein
CaMKII: calcium/calmodulin-dependent protein kinase
cAMP: cyclic AMP
DMSO: dimethyl sulfoxide
GluA: glutamate receptor A
fEPSP: field excitatory postsynaptic potential
L-SPD: L-Stepholidine
LTP: long-term potentiation
PKA: protein kinase A
PS1: presenilin 1
THPB: tetrahydroprotoberberine
