# Supplementary figures and images for: L-Stepholidine rescues memory deficit and synaptic plasticity in models of Alzheimer's disease via activating dopamine D1 receptor/PKA signaling pathway
Source: Cell Death Dis. 2015 Nov 5;6(11):e1965–. doi: 10.1038/cddis.2015.315 (PMC4670924; doi:10.1038/cddis.2015.315)

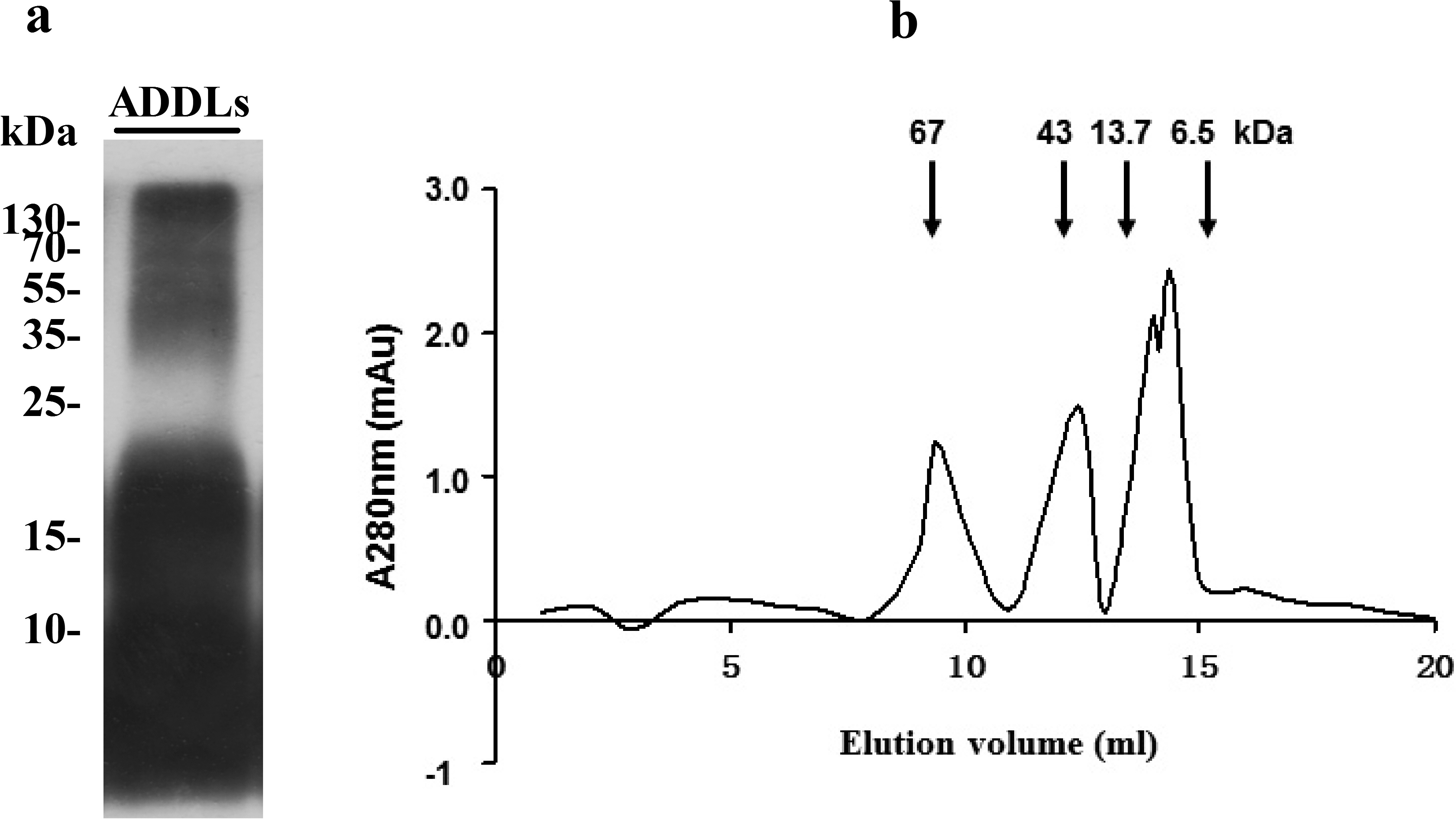

Supplement: Supplementary Figure 1 [file cddis2015315x1.tif]

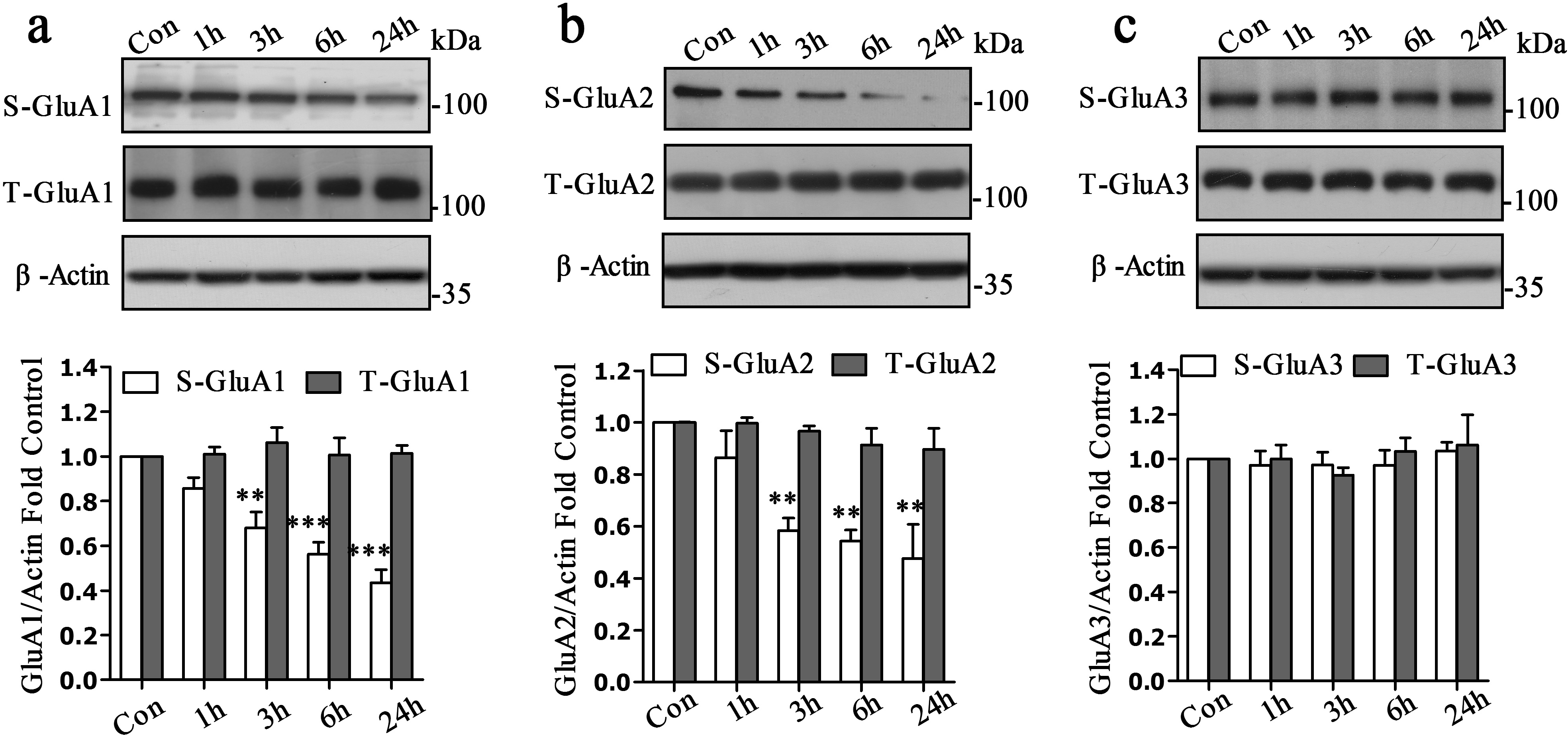

Supplement: Supplementary Figure 2 [file cddis2015315x2.tif]
