# Supplementary material for: L-Stepholidine rescues memory deficit and synaptic plasticity in models of Alzheimer's disease via activating dopamine D1 receptor/PKA signaling pathway
Source: Cell Death Dis. 2015 Nov 5;6(11):e1965–. doi: 10.1038/cddis.2015.315 (PMC4670924; doi:10.1038/cddis.2015.315)
Supplement: Supplementary Figure Legends [file cddis2015315x3.doc]

**Supplementary Material and Methods**

**Cell surface protein labeling and detection.** Cell surface proteins were labeled and isolated using Cell Surface Protein Isolation kit (Thermo Scientific) according to the manufacturer’s instruction. Briefly, the cells were washed in ice-cold phosphate buffered solution (PBS) and incubated in EZ-Link™ Sulfo-NHS-SS-Biotin solution (0.5 mg/ml, dissolved in PBS, pH 8.0) at 4°C for 30 min.  Cells were washed three times with ice-cold PBS (pH 8.0) to remove non-reacted biotinylation reagent. Cells were then homogenized in modified RIPA buffer [50 mM Tris-Cl (pH 7.4), 0.25% Na deoxycholate, 150 mM NaCl, 1 mM EDTA, 50 mM noctylglucoside] supplemented with protease inhibitors. Homogenized cells were incubated under constant rotation at 4°C for 30 min and then centrifuged at 200 g for 5 min. After centrifugation, the pellet was discarded and the protein concentration was measured. Equal amounts of protein were incubated under constant rotation with NeutrAvidin® Agarose Resins beads for 1h at room temperature. Protein extracts were separated on SDS-PAGE gels and transferred to PVDF membranes. Proteins were detected by Western blot analysis.

**Size exclusion chromatography.** The Superdex 75 10/300 GL column (Amersham Biosciences AB, Uppsala, Sweden) was used on an automated AKTA pure system (GE Healthcare) at a flow rate of 0.8 ml/min. The column is capable of resolving proteins below 70 kDa. The column is equilibrated with 1.5 column volumes of buffer (0.05 M phosphate, pH 7.2, and 150 mM NaCl). 0.5 ml ADDLs (0.2 mg/ml) was loaded onto the column. The approximate molecular weight of the Aβ oligomers were estimated by using standard globular proteins including BSA (67 kDa), ovalbumin (43 kDa), ribonuclease A (13.7 kDa), and aprotinin (6.5 kDa).

**Figure Legends**

**Figure S1 Aggregation profile of ADDLs by Western blot analysis and size exclusion chromatography. (a)** Freshly prepared ADDLs (500 nM) migrated on 15% SDS-PAGE. **(b)** Typical chromatogram of a size exclusion chromatography. Four distinct size ranges were observed.

**Figure S2 Biotinylation assays revealed a significant decrease of surface expression of GluA1/GluA2 in cultured hippocampal neurons.** Surface expression of GluA1 (S-GluA1) and GluA2 (S-GluA2) were significantly decreased after treating with ADDLs (500 nM) for 3-24 hours, while surface expression of GluA3 (S-GluA3) was not changed. Total expression of GluA1 -3 (T-GluA) were not changed. (*n* = 3 in each group). ^**^ *P* < 0.01, ^***^ *P* < 0.001 vs corresponding control group (Con). Data are represented as mean ± SEM.
